# The ACT Predicts Academic Performance—But Why?

**DOI:** 10.3390/jintelligence11010009

**Published:** 2023-01-03

**Authors:** Alexander P. Burgoyne, Kelly M. Stec, Kimberly M. Fenn, David Z. Hambrick

**Affiliations:** 1 School of Psychology, Georgia Institute of Technology, Atlanta, GA 30332, USA; 2 Michigan Public Health Institute, Okemos, MI 48864, USA; 3 Department of Psychology, Michigan State University, East Lansing, MI 48824, USA

**Keywords:** ACT, personality, academic achievement, general intelligence

## Abstract

Scores on the ACT college entrance exam predict college grades to a statistically and practically significant degree, but what explains this predictive validity? The most obvious possibility is general intelligence—or psychometric “*g*”. However, inconsistent with this hypothesis, even when independent measures of *g* are statistically controlled, ACT scores still positively predict college grades. Here, in a study of 182 students enrolled in two Introductory Psychology courses, we tested whether pre-course knowledge, motivation, interest, and/or personality characteristics such as grit and self-control could explain the relationship between ACT and course performance after controlling for *g*. Surprisingly, none could. We speculate about what other factors might explain the robust relationship between ACT scores and academic performance.

## 1. Background

Every year, millions of high school students seeking admission to U.S. colleges and universities take the SAT and/or ACT. These tests have their critics. Writing in the *New York Times*, the academic Jennifer Finney Boylan (2014) called the use of the SAT to make college admissions decisions a “national scandal”. More recently, policy changes have followed suit, with some universities abolishing the use of standardized test scores in admissions (Lorin 2022). Nevertheless, the SAT and ACT yield scores that predict performance in the college classroom. Correlations between scores on the tests and college grade point average (GPA) are typically in the .30–.50 range (Kuncel and Hezlett 2007; Sackett et al. 2009; Schmitt et al. 2009). 

What explains this predictive validity? The most obvious possibility is general intelligence—or psychometric “*g*”—which is highly predictive of academic performance (Deary et al. 2007). After all, the ACT and SAT are themselves tests of cognitive ability, and scores on the tests correlate highly with independent estimates of *g*. For example, in a sample of 1075 college students, Koenig et al. (2008) found a correlation of .77 between ACT scores and a *g* factor extracted from the Armed Services Vocational Aptitude Battery (see also Frey and Detterman 2004). 

As much sense as this *g hypothesis* makes, it may not be entirely correct. In both university and nationally representative samples, Coyle and Pillow (2008) found that although both the SAT and ACT were highly *g* loaded (factor loadings = .75 to .92), the tests predicted GPA after statistically controlling for *g.* Specifically, with a latent *g* factor comprising either test and independent measures of cognitive ability (e.g., Wonderlic scores), residual terms for SAT and ACT, reflecting non-*g* variance, positively predicted GPA. In fact, in 3 of 4 models, the non-*g* effects were similar in magnitude to the zero-order correlations of SAT and ACT with GPA, indicating *g* played a somewhat minor role in explaining the relationship between scores on the tests and GPA.

Before proceeding, we note one limitation of Coyle and Pillow’s investigation. The outcome variable in their studies was college GPA rather than grade in a single course. GPA can be difficult to interpret across individuals who have taken different courses. For example, earning a 4.0 in introductory physics probably requires a higher level of cognitive ability than a 4.0 in introductory psychology. 

If *g* does not explain the predictive validity of college entrance exams, what does? Coyle and Pillow (2008) suggested that, in addition to scholastic skills, these tests may capture personality traits that relate to academic performance. Here, using performance in a single course, Introductory Psychology, we tested Coyle and Pillow’s (2008) hypothesis, focusing on personality traits that have been shown to correlate with academic performance. We considered two “big-five” traits. *Conscientiousness* (C) is characterized by need for achievement and commitment to work (Costa and McCrae 1992), and *openness* (O) by a tendency to seek out new experiences (McCrae and Costa 1997). We also considered two “character” traits. *Self-control* refers to the capacity to interrupt and override undesirable behaviors (Tangney et al. 2004), whereas *grit* is defined as persistence toward long-term goals (Duckworth and Gross 2014). 

These personality and character traits could influence performance in any academic course (for reviews, see Trapmann et al. 2007; Richardson et al. 2012). We also considered course-specific factors: motivation, interest, pre-course knowledge, and studying. Motivation to succeed in a course and interest in its content predict a range of behaviors related to success such as studying, paying attention in class, taking notes, etc. (Lee et al. 2014; Singh et al. 2002), while prior knowledge of a topic facilitates new learning by providing a structure for comprehending and integrating new information about that topic (Hambrick et al. 2010; Yenilmez et al. 2006). 

Any (or all) of the preceding factors may covary with ACT scores. For example, students who attend elite, well-funded high schools may have intensive ACT preparation and may also have had the opportunity to take a wider range of courses, leading to higher levels of motivation, interest, and pre-course knowledge for various subjects once they enter college, compared to students from other high schools. This may be especially true for non-core subjects such as psychology, which is not taught at all high schools. Along with having the opportunity for ACT preparation, students who attend top high schools may also develop stronger study skills than other students.

### Research Question

The major goal of this study was to understand what accounts for the predictive validity of ACT scores for grades in an Introductory Psychology course. Near the beginning of a semester, we asked participants for permission to access their ACT scores through the university and had them complete tests and questionnaires to measure cognitive ability, personality, interest, motivation, and pre-course knowledge of psychology. At the end of the semester, the participants completed a post-course test. In a series of exploratory regression and structural equation analyses, we tested for effects of the ACT on course performance, before and after controlling for *g* and the aforementioned factors.

## 2. Method

### 2.1. Participants

Participants were 193 students from two sections of an introductory psychology course at Michigan State University, taught by two different instructors (authors of this article). Introductory Psychology is a popular course at this university, attended by psychology majors as well as non-majors. Typically, around 50–60% of students are freshmen, and around 50% or less of the students are psychology majors. In our sample, eleven participants were excluded because they did not consent for their ACT scores to be used in analyses, leaving a final sample of 182 participants (129 female, 53 male; *n* = 70 for Section 1, *n* = 112 for Section 2) who ranged in age from 18 to 22 (*M* = 18.7, *SD* = .9). All participants were native English speakers and received credit towards their required participation in research for the course. 

We set out to test as many participants as possible within a semester. Our sample size is typical for individual-difference research and provides adequate post hoc statistical power to detect small-to-medium correlations (e.g., *r* = .20, 1 − β = .78). 

### 2.2. Materials 

#### 2.2.1. Study Habits Questionnaire

In this questionnaire, participants were asked questions about how they studied for Introductory Psychology (“regular study time”), and how they studied specifically for the first test of the semester (“test study time”). For each, they were asked to give a single weekly time estimate (e.g., 10 h), including how much of that time was spent “alone in a quiet environment, free of noise and other distractions such as texting, cell phones, television, etc.”. They were also asked to indicate the number of days they studied, and respond to a yes/no question about whether they used a calendar or planner to schedule their study time. 

#### 2.2.2. Cognitive Ability Tests

To estimate *g*, we had participants complete four paper-and-pencil cognitive ability tests. The first two were tests of “fluid” ability (Gf) and the latter two were tests of “crystallized” ability (Gc). In *letter sets* (Ekstrom et al. 1976), participants were instructed to find which series of four letters did not follow the same pattern as the other four options. They were given 7 minutes to complete 15 items, each containing five options (four that followed the pattern and one that did not—the correct answer). In *series completion* (Zachary and Shipley 1986), participants were instructed to figure out the final letters or numbers that completed a logical sequence. They were given 4 minutes to complete 20 items. Answers ranged from one to five characters and were either all letters or all numbers in each trial. In *vocabulary* (Zachary and Shipley 1986), participants were instructed to circle the synonym to a given word. They were given 4 minutes to complete 15 items, each with four multiple choice answers. In *reading comprehension* (Kane et al. 2004), participants were instructed to choose the answer that best completed the meaning of short paragraphs. They were given 6 min to complete 10 items that had five multiple choice answers. For each cognitive ability test, the score was the number correct.

#### 2.2.3. Personality Scales

All personality scales were administered in a paper-and-pencil format. Participants responded on a 5-point Likert scale from “Very Much Like Me” to “Not Like Me at All” and the score for each scale was the sum of ratings across items. There was no time limit.

***Big five traits.*** We used the 20-item “mini” International Personality Item Pool (IPIP) inventory (Donnellan et al. 2006) to measure the big-five personality traits (neuroticism, extraversion, openness, agreeableness, and conscientiousness). In addition, because conscientiousness was a prime candidate to mediate the ACT-grade relationship, we administered 60 items from the IPIP (Goldberg 1999) to measure the six facets of conscientiousness (self-efficacy, orderliness, dutifulness, achievement-striving, self-discipline, and cautiousness); there were 10 items per facet. 

***Self-control.*** We used a 13-item scale developed by Tangney et al. (2004) to assess self-control (e.g., “I often act without thinking through all the alternatives”—reverse), along with the 19-item Adult Temperament Questionnaire (Evans and Rothbart 2007) to measure three facets of effortful control: attentional (capacity to focus or shift attention as required; e.g., “When interrupted or distracted, I usually can easily shift my attention back to whatever I was doing before”), activation (capacity to perform an action when there is a strong tendency to avoid it, e.g., “I can keep performing a task even when I would rather not do it”), and inhibitory control (capacity to suppress inappropriate behavior; e.g., “It is easy for me to hold back my laughter in a situation when laughter wouldn’t be appropriate”). A self-control variable was created by taking the average of the scores on these scales.

***Grit.*** We used the 12-item Short Grit Scale (Duckworth and Quinn 2009) to measure grit. Half of the items were positively worded (e.g., “I have overcome setbacks to conquer an important challenge”), and half were negatively worded (e.g., “My interests change from year to year”). 

### 2.3. Procedure

Within 1 week of the first test of the semester, participants reported to the lab for the study. Participants were asked to provide consent for researchers to access their ACT scores through the Office of the Registrar and their course grades through their instructors. Participants were seated at tables in a seminar room and given a packet containing the Study Habits Questionnaire, Letter Sets, Vocabulary, Series Completion, and Reading Comprehension (in that order). Finally, all participants completed the personality scales. Participants were then debriefed and dismissed from the lab. Participants were tested in groups of up to 30 individuals at a time.

#### Course Performance 

On the first day of class, participants completed a 50-question test designed by the course professors to measure students’ knowledge of psychology; we refer to the score on this test as *pre-course knowledge.* The questions covered the following areas (with the number of questions in parentheses): introduction and history (4); research methods (4); the brain and behavior (3); sensation and perception (3); consciousness and sleep (3); development (4); heredity and evolution (3); learning (3); memory (4); and language and thought (3); intelligence (3); personality (3); emotion and motivation (3); social psychology (4); and psychological disorders and psychotherapy (3). The questions were in the same order for all participants. During the semester, participants completed four non-cumulative tests; we refer to the average of scores on these tests as *test average.* Then, as the cumulative final exam in each course, the 50-question test of pre-course knowledge was again administered on the last day of class; we refer to score on this test as *post-course knowledge.* The question format was multiple-choice (4-alternative) and the score was the percentage correct. 

### 2.4. Data Preparation

We screened the data for univariate outliers (values more than 3.5 *SD*s from sample means); there were 7 outliers, which we winsorized to 3.5 *SD*s from the sample means. Data are openly available at: https://osf.io/6yagj/ (accessed on 16 May 2019). We report all data exclusions, manipulations, measures, and analyses. This study was not preregistered.

## 3. Results

Descriptive statistics are presented in Table 1 for the two Introductory Psychology sections; correlations are in Table 2 and Table 3. Scores on the ACT correlated positively with cognitive ability (avg. *r* = .45), particularly the crystallized intelligence measures (avg. *r* = .49), which correlated positively with course performance. 

As expected, the measures of cognitive ability correlated positively with each other (Table 2), implying the existence of a *g* factor. Supporting this inference, we entered the cognitive ability variables into an exploratory factor analysis (principal axis), and the variables had strong positive loadings on the first unrotated factor, ranging from .53 to .60. We saved the score for this factor for use as the estimate of *g* in the regression analyses reported next. Replicating previous findings (e.g., Frey and Detterman 2004; Koenig et al. 2008), this *g* factor correlated highly (all *p*s < .001) with ACT scores, both overall (*r* = .65) and in each section, Section 1 (*r* = .57) and Section 2 (*r* = .67).

### 3.1. Regression Analyses Predicting Test Average

In a series of regression analyses, we estimated the incremental contribution of ACT to test average before and after controlling for *g* and potential mediator variables. We analyzed the data separately by course section, given that all but the last test (i.e., the post-course knowledge test) were different across the sections. 

We evaluated three models. In Model 1, we regressed test average onto ACT. In Model 2, we regressed test average onto *g* (Step 1) and ACT (Step 2). In Model 3, with a separate analysis for each potential mediator, we regressed test average onto *g* (Step 1), a mediator variable (Step 2), and ACT (Step 3).^1^ The question of interest was whether (a) ACT would explain variance in test average above and beyond *g*, and (b) if so, whether statistically controlling for each of the mediators would reduce this incremental contribution of ACT to test average.

Results are summarized in Table 4. ACT explained a sizeable amount of the variance in test average in both sections: Section 1 (*R*^2^ = .27, *p* < .001) and Section 2 (*R*^2^ = .25, *p* < .001). Moreover, in both sections, ACT added significantly to the prediction of test average after controlling for *g:* Section 1 (Δ*R*^2^ = .21, *p* < .001) and Section 2 (Δ*R*^2^ = .19, *p* < .001). However, in neither section did any of the mediator variables substantially reduce this incremental contribution of ACT. That is, in Model 3, the effect of ACT on test average remained statistically significant in all analyses (all *p*s < .001). 

It is also worth noting that, alone, *g* was a significant predictor of test average in both samples: Section 1 (β = .25, *R*^2^ = .06, *p* = .035) and Section 2 (β = .27, *R*^2^ = .07, *p* = .004). However, as shown in Table 4, its effects were no longer significant with ACT added to the model. This finding adds to the case that the predictive validity of the ACT for course performance in our sample was driven by one or more factors unrelated to *g.* To put it another way, the ACT appears to capture one or more factors predictive of course performance that tests of cognitive ability miss.

#### 3.1.1. Study Time

We also examined whether amount of time spent studying for Test 1 mediated the relationship between ACT and grade on Test 1. The outcome variable was the score on Test 1. In Step 1 we added *g*, in Step 2 we added test study time, and in Step 3 we added ACT. In both sections, ACT was still a significant predictor of Test 1 score after accounting for study time and *g* (*p*s ≤ .003). The effect of study time on Test 1 score was not significant (*p*s > .31). 

#### 3.1.2. ACT Subtests

ACT may have predicted course performance because some of the subtests capture knowledge directly relevant to success in the course. For example, the Natural Science subtest includes questions to assess test takers’ ability to read and interpret graphs, which would be beneficial in Introductory Psychology. To investigate this possibility, we regressed the ACT subtest scores onto course performance. The results are displayed in Table 5 in terms of the overall *R*^2^ and unique *R*^2^s (i.e., the squared semi-partial *r*s), reflecting the independent contributions of the ACT subtests to the prediction of test average. The unique *R*^2^ for ACT-English was statistically significant in Section 2 (unique *R*^2^ *=* .07, β = .37, *p* = .002) but was non-significant in Section 1 (unique *R*^2^ *=* .03, β = .28, *p* = .089). However, the unique *R*^2^ for the Natural Science subtest was near zero and non-significant in both course sections (i.e., unique *R*^2^ values of .02 and .01). Note also that the overall *R*^2^ in each section was much larger than the sum of the unique *R*^2^s, further indicating that the relationship between overall ACT score and test average was driven by factors measured by all the subtests rather than to knowledge captured by particular subsets. 

### 3.2. Structural Equation Models Predicting Post-Course Knowledge

Next, following Coyle and Pillow’s (2008) data-analytic approach, we used structural equation modeling (SEM) with maximum likelihood estimation to evaluate the effect of ACT on the post-course knowledge test, controlling for *g.* Prior to conducting this analysis, we tested whether any of the predictor variables interacted with course section (i.e., Section 1 or Section 2) to predict post-course knowledge. Only 1 of 13 interactions was statistically significant (Openness to Experience × Class Section; β = .22, *p* = .005). Thus, to maximize statistical power, we combined data from the two sections for use in the SEM. (Recall that the same post-course exam was used in both sections; we could therefore collapse across sections. We elected not to perform SEM with test average as the outcome variable because the tests were different across sections, and the sample sizes per section would not provide sufficient statistical power and precision for the SEMs.)

**Figure 1:**
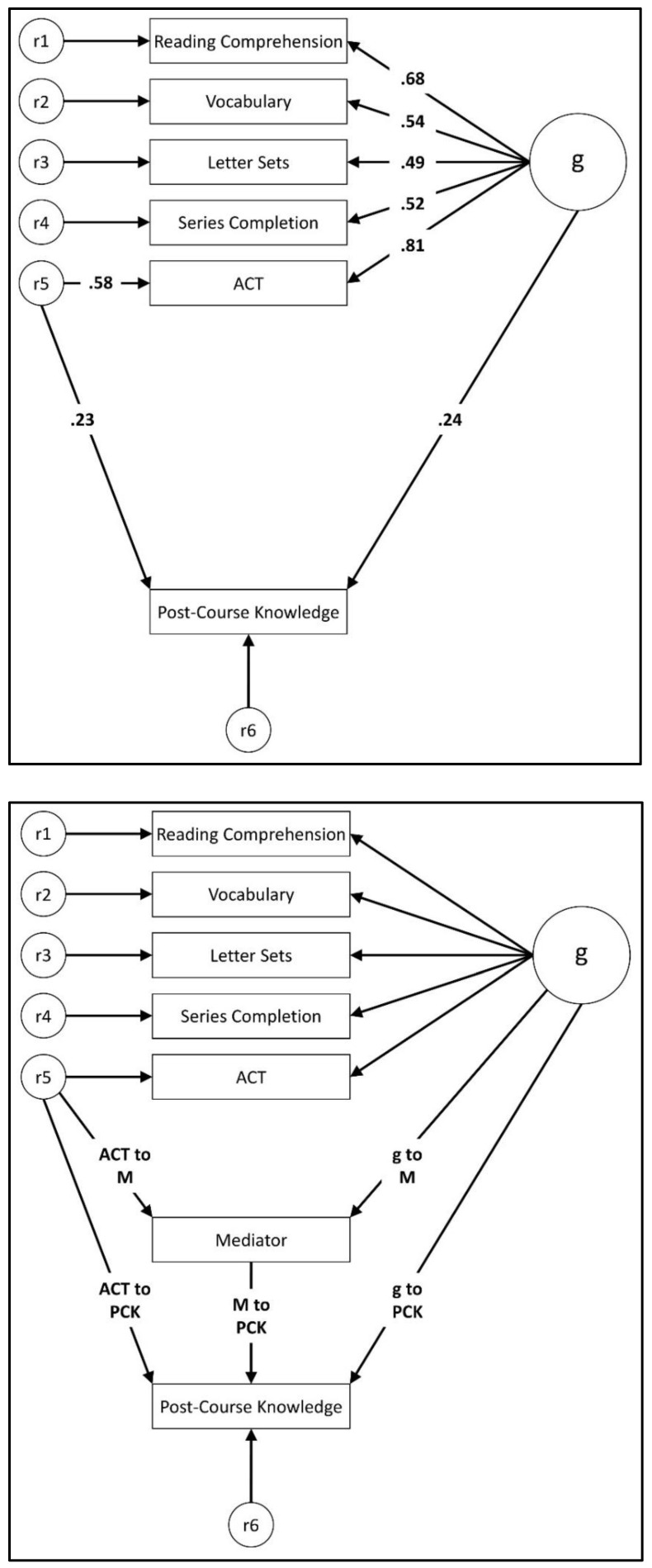
Top panel: SEM with *g* and the ACT residual predicting post-course knowledge (PCK). Bottom panel: General mediation model used to test whether the ACT residual-academic performance relationship is accounted for by a mediator variable. See Table 6 for parameter estimates for the mediation models.

Two steps were involved in the SEM. First, we created a structural model that included (a) a *g* factor, with loadings on the cognitive ability variables (Reading Comprehension, Vocabulary, Letter Sets, Series Completion) as well as ACT, and (b) a unidirectional path from the ACT residual term (i.e., error term) to post-course knowledge (see Figure 1: top panel). Second, we tested whether any of the personality, motivation, interest, or pre-course knowledge variables mediated the relationship between the ACT residual and post-course knowledge, conducting a separate analysis for each potential mediator (see Figure 1: bottom panel).^2^ The question of interest was whether the indirect path from the ACT residual through the mediator to post-course knowledge was statistically significant (Hayes 2009). 

As expected, *g* had a statistically significant positive effect (β = .24, *p* = .008) on post-course knowledge. Students with a high level of *g* tended to do better on the post-course knowledge test than students with a lower level of *g.* More importantly, however, the effect of the ACT residual on post-course knowledge (β = .23, *p* = .023) was also statistically significant, even though ACT had a very high *g* loading (.81). Thus, irrespective of their estimated level of *g,* participants who did well on the ACT tended to do better on the post-course knowledge test than did those who scored lower on the ACT. This finding replicates Coyle and Pillow’s (2008) results.

With this established, we tested a series of mediation models to determine whether the relationship between the ACT residual and post-course knowledge was mediated through pre-course knowledge, personality, course motivation, and/or course interest. In each analysis, we added unidirectional paths from the *g* factor and the ACT residual to the hypothesized mediator variable. We then added a predictor path from the mediator to post-course knowledge. For each analysis, the question of interest was whether the indirect path from the ACT residual (i.e., error term) through the mediator to post-course knowledge was statistically significant, as determined by bootstrap analyses (see Hayes 2009).

Parameter estimates for the specific mediation models we tested are presented in Table 5. As can be seen, inclusion of the mediators in the model had very little impact on the path from the ACT residual to post-course knowledge. That is, across the models, the path coefficient for the ACT residual was almost the same before adding the mediators to the model (β = .23) as it was after doing so (Mean β = .22, range = .19 to .25). Consistent with this impression, the bootstrap analyses revealed that in no case was the indirect path from the ACT residual through the mediator to post-course knowledge statistically significant (all *p*s > .05). Taken together, the results indicate that the contribution of non-*g* variance in ACT scores to academic performance was not attributable to pre-course knowledge, conscientiousness, openness, self-control, grit, or course interest. 

One other result from the SEM is noteworthy. Pre-course knowledge fully mediated the relationship between the *g* factor and post-course knowledge (95% bias corrected bootstrap confidence interval [based on 5000 bootstrap samples] for the indirect effect = .07 to .26, *p* < .001). After adding pre-course knowledge to the model as a mediator, the direct path from *g* to post-course knowledge was no longer statistically significant (β = .11, *p* = .277), whereas the path from *g* to pre-course knowledge (β = .44, *p* < .001) and the path from pre-course knowledge to post-course knowledge (β = .31, *p* < .001) were statistically significant. The model accounted for 18.1% of the variance in post-course knowledge.

## 4. Discussion

Scores on college entrance exams predict college grades, but why? The most obvious possibility is general intelligence (*g*). However, consistent with earlier findings (Coyle and Pillow 2008), we found that ACT scores predicted academic performance even after statistically controlling for an independent assessment of *g.* Somewhat embarrassingly, a few years ago, the last author of this article overlooked Coyle and Pillow’s article and suggested that it must be *g* that explains the validity of the SAT (Hambrick and Chabris 2014). Scores on college entrance exams correlate very highly with *g* (Frey and Detterman 2004; Koenig et al. 2008)—but *g* may *not* be what explains that predictive validity of the tests. 

In this study, using Introductory Psychology as the venue for our research, we found that the ACT-course performance relationship remained significant (and almost unchanged) after controlling for personality, interest, motivation, and pre-course knowledge. This was true for an outcome variable reflecting the average score on tests taken during the semester, as well as on one reflecting performance on a post-course knowledge test. Interestingly, in the regression analyses, *g* was not a significant predictor of semester test average with ACT in the model, whereas in the SEM, both *g* and ACT had significant effects on post-course knowledge. One possible explanation for this finding that *g* was a significant unique predictor of post-course knowledge but not test average is that the cumulative post-test required that students have mastered more information at once than did each test given during the semester, placing a greater demand on general cognitive ability. However, with respect to our research question, what is more important is that in both analyses (a) ACT predicted the outcome variable, and (b) *g* did not account for this effect of ACT. 

So, we once again ask: If *g* does not fully explain the predictive validity of the ACT, what does? Specifically, what explains the ACT-course performance relationship we observed? One possibility is course-relevant knowledge/skills. The ACT captures a broader range of knowledge/skills than the tests we used to measure *g*, some of which may be directly applicable to learning content in introductory psychology. Stated differently, the ACT may capture knowledge acquired through years of schooling, some of which may be relevant to psychology and therefore provide scaffolding that facilitates the acquisition of new domain-specific knowledge. However, there is no support for this transfer-based explanation in our data. The Natural Science subtest of the ACT captures knowledge/skills that are potentially relevant in Introductory Psychology (e.g., how to read graphs), whereas almost no math is required. However, as it was for the Mathematics subtest, the unique *R*^2^s for the Natural Science subtest were near zero (Table 5). 

Another possibility is *college preparedness.* Students who attend rigorous, well-funded high schools may arrive at college with a savviness that helps them succeed. We found no evidence that amount of studying mediated the ACT-performance relationship, but the quality of studying may be more critical. For example, students who test themselves while studying may perform better on exams than students who simply re-read course materials (Butler 2010). 

Socioeconomic variables are important to consider, too. There is a robust relationship between high school quality and socioeconomic status: Students who attend top high schools tend to be from affluent families (Currie and Thomas 2001). Once they get to college, these students should have greater financial resources for succeeding. For instance, they are less likely to need to work during college to support themselves, and more likely to be able to afford tutors, textbooks, and computers. Sackett et al. (2009) found that controlling for parental SES had minimal impact on the relationship between SAT scores and college grades. However, as a more direct test of the role of resources in the ACT-performance relationship, it would be worthwhile to ask participants to report how much money they have for academic-related expenses. 

Finally, it is important to point out that the ACT and tests taken in a college course are extremely important from the students’ perspective. A student’s performance on these “high stakes” tests has a direct impact on their future. By contrast, little is at stake with cognitive ability tests taken in the laboratory for a psychological study; participants are not even told their scores. Thus, the ACT and college tests may be thought of as tests of “maximal performance”, whereas lab tests may more reflect “typical performance” (Ackerman and Kanfer 2004). A high-stakes testing situation could activate a number of factors that could explain the correlation between ACT and course performance, including focused attention, achievement motivation, and test anxiety, to name a few. It would be difficult to recreate an equally high-stakes testing situation in the lab, but this could be an avenue for future research. It is possible that monetary incentives could activate some of these factors. On a related note, test-taking skill (especially skill in guessing on multiple-choice tests) may influence the ACT-grade correlation, although to some degree the tests we used to measure cognitive ability may have captured this factor.

The analyses further revealed that pre-course knowledge, while not explaining the ACT-post-course knowledge relationship, mediated the relationship between *g* and post-course knowledge. That is, once pre-course knowledge was entered into the model, the direct relationship between *g* and post-course knowledge was no longer significant. This finding is consistent with the finding from the job performance literature showing that the effect of *g* on job performance is mediated through job knowledge (Schmidt et al. 1988). People who have a high level of cognitive ability acquire more knowledge through experience than people with a lower level of cognitive ability.

### 4.1. Limitations

We note a few limitations of our study. First and foremost, our conclusions are limited by our sample and by our selection of tests to measure *g.* Our sample was relatively modest in size, with 182 students represented in the structural equation analyses and fewer students represented in analyses at the observed level (e.g., correlations and regression analyses) due to sampling two distinct introductory psychology course sections. Thus, our ability to detect small or very small effects was reduced by our statistical power. Furthermore, the range of cognitive ability in our sample was restricted; the standard deviation for overall ACT score was 3.2 in our sample, compared to 5.6 for all high school students who take the test (ACT Technical Manual 2017). Also, the reliability of our composite *g* measure was somewhat low (.65)*,* and we only used four tests of cognitive ability to estimate *g* (i.e., two Gf and two Gc tests). A broader set of cognitive ability tests would allow for a better estimate of general intelligence and the relationship between general intelligence and ACT performance. Thus, it is safe to assume that we underestimated the ACT-*g* correlation in our study. That is, ACT is probably more *g*-saturated than our results indicate. 

At the same time, the *g* loading for ACT (.81) in our sample is in line with *g* loadings for ACT and SAT (.75–.92; avg. = .84) reported by Coyle and Pillow (2008), who used a greater number of cognitive ability tests to measure *g* and tested samples representing wider ranges of cognitive ability. Furthermore, even if the correlation between ACT and *g* is corrected for measurement error and range restriction, there is still statistical “room” for a non-*g* effect on course performance. Using the earlier reported reliability estimates for ACT (.85) and *g* (.65), the correlation between the variables increases from .65 to .87 after correction for unreliability. In turn, using the earlier noted *SD*s for ACT in our sample (3.2) versus the national sample (5.2), this correlation increases to .95 after correction for direct range restriction in ACT. Although this correlation is very strong, squaring it reveals that about 10% of the variance in ACT is independent of *g* [i.e., (1 – .95^2^) × 100 = 9.75%]. Coyle and Pillow’s (2008) Study 1 provides a further illustration of this point: SAT had a *g* loading of .90, and yet the SAT residual still had an effect of .36 on GPA. 

Taken together, these observations argue against an interpretation of the results which holds that the sole reason non-*g* variance in ACT performance predicted course performance (i.e., post-course knowledge) in our study is because of psychometric limitations. To put it another way, even if the *g* loading for ACT were substantially higher than what we observed in this study, it is still possible that there would have been significant non-*g* effects of ACT on the course performance outcomes. A predictor variable can be highly *g*-loaded, but still have an effect on an outcome variable independent of *g.*

We further note that the results may differ by course. As already mentioned, introductory psychology is probably less cognitively demanding than, say, introductory physics. Psychometric *g* may well account for the predictive validity of the ACT in more demanding courses. As a final limitation, we had only limited data on study behavior (a single test). It is conceivable that study behavior at least partly explains the relationship between ACT and academic performance. In future studies, we will examine this possibility by collecting detailed information on how much time students spend studying and the quality of this study time. 

### 4.2. Future Directions

In the 2019 college admissions cheating scandal, dozens of parents were alleged to have paid large sums of money to have a “ringer” take the ACT for their children, or to have their children’s test forms altered to increase their scores. This is not an indictment of the ACT, but rather a sobering reminder of the importance of scores on college entrance exams in our society. All else equal, a high school student who gets a high score on the SAT or ACT will have a greater opportunity to attend a top university or college than a student who gets a lower score. Graduating from such an institute may translate into greater opportunities in life—beginning with getting a good job. As one rather obvious example, average SAT/ACT scores for students admitted to Ivy League universities such as Princeton, Harvard, and Yale are typically above the 95th percentile (National University Rankings). An Ivy League diploma does not guarantee success in life, but as Department of Education’s College Scorecard Data (n.d.) reveal, the median income for an Ivy League graduate is more than twice that for graduates of other institutions (Ingraham 2015). 

From a fairness perspective,^3^ it is critical to understand what explains the predictive validity of college entrance exams. There is no doubt that these tests measure skills important for success in the college classroom, such as verbal ability and mathematical ability. However, it would be concerning if factors reflecting differential opportunity influenced the predictive validity of the tests. Presumably with this in mind, the College Board announced that, along with a student’s SAT score, it will report to colleges an “adversity score” based on 15 variables, ranging from quality of a student’s high school to the average income and crime rate in the neighborhood where they live (Hartocollis 2019). From our perspective, it will be especially interesting whether this adversity score explains the *g*-partialled relationship between ACT scores and academic performance. 

Our goal for future research is to investigate the ACT-course performance relationship in larger and more representative samples, using larger batteries of cognitive ability to assess *g*, and across a broad range of academic courses. We also plan to assess more potentially relevant predictors of course performance. Following up on other work by Coyle and colleagues (Coyle et al. 2015), we will investigate how non-*g* variance in ACT scores predicts performance across different types of courses. The findings from this research will increase understanding of factors contributing to the predictive validity of college entrance exams and help ensure that the tests are used fairly. 

## Figures and Tables

**Table 1:** Descriptive Statistics.

			Section 1			Section 2		
Variable	Items	Rel.	*N*	*M*	*SD*	*N*	*M*	*SD*
**Cognitive ability**								
Letter sets	15	.68	70	9.97	2.92	112	11.08	2.13
Series completion	20	.53	70	14.63	1.78	112	14.87	1.84
Vocabulary	15	.62	70	8.57	2.51	112	9.41	2.44
Reading comprehension	10	.62	70	4.90	1.99	112	5.37	2.36
**Personality**								
Conscientiousness	4	.71	70	8.77	2.79	112	9.25	3.11
Dutifulness	10	.78	70	18.14	4.33	112	18.16	4.66
Cautiousness	10	.85	70	28.01	6.44	112	26.38	6.87
Self-efficacy	10	.79	70	21.23	4.55	112	21.01	5.11
Achievement striving	10	.82	70	20.34	4.82	112	20.83	5.89
Self-discipline	10	.87	70	25.60	5.99	112	26.52	6.88
Orderliness	10	.82	70	24.61	6.66	112	25.37	6.28
Openness	4	.73	70	9.19	2.66	112	9.47	2.99
Self-control	32	.87	70	22.60	3.53	112	22.15	4.00
Grit	12	.78	70	31.61	6.17	112	31.31	6.81
Course interest	1	-	70	2.86	0.82	112	3.02	0.73
Course motivation	1	-	70	3.34	0.70	112	3.43	0.63
**Course Performance**								
Pre-course knowledge	50	.59	68	39.59	8.89	112	44.45	8.96
Post-course knowledge	50	.68	70	80.11	11.00	111	79.05	9.26
Test average	4 *	.82	70	78.37	11.35	112	82.95	6.93
**ACT**								
Overall score	-	.85/.97	70	23.24	2.88	112	24.97	3.29
English	-	.92	70	23.67	3.60	112	24.93	4.16
Mathematics	-	.91	70	22.93	3.22	112	24.44	3.70
Reading	-	.87	70	22.89	4.34	112	25.59	4.41
Natural Science	-	.85	70	22.84	2.69	112	24.40	3.52

Note. Rel., reliability estimate. Coefficient alphas computed using the total sample for the cognitive ability and personality variables. For overall ACT score, the left value is a coefficient alpha computed from the subtest scores and the right value is the alpha reported in the ACT Technical Manual (2017); for the ACT subtests, the coefficient alphas are from the manual. Multiple *R*s for the pre-course knowledge and post-course knowledge scores (obtained by regressing each variable onto the other variables in the data set). * There were four 50-item tests; scores on these tests were used to compute the coefficient alpha for test average.

**Table 2:** Correlation Matrix.

	1	2	3	4	5	6	7	8	9	10	11	12	13	14
(1) ACT	–	**.37**	**.44**	**.44**	**.64**	.15	.10	−.14	.09	.16	.02	**.36**	**.28**	**.50**
(2) Letter sets	**.36**	–	**.56**	**.25**	**.32**	−.01	.11	−.05	−.05	.03	−.05	.09	.10	.08
(3) Series completion	**.38**	**.43**	–	**.25**	**.31**	.00	.06	.03	.13	.01	−.04	.17	.12	.16
(4) Vocabulary	**.28**	−.01	.13	–	**.52**	.14	−.02	.13	.18	−.03	−.01	.17	.05	.18
(5) Reading comprehension	**.34**	.13	.08	**.42**	–	.13	.07	−.01	.08	.18	−.05	**.29**	**.22**	**.32**
(6) Conscientiousness	.01	.00	.00	.09	−.18	–	−.03	**.49**	**.56**	.07	−.04	.03	.02	.08
(7) Openness	.08	.12	−.15	**−.25**	−.01	.13	–	.12	−.04	−.11	.01	−.01	−.11	.03
(8) Self-control	.01	.01	.00	−.16	.00	**.42**	**.25**	–	**.69**	−.10	−.05	−.01	−.03	−.06
(9) Grit	.17	.09	.01	.05	.04	**.33**	.15	**.57**	–	−.13	−.17	.02	−.02	.01
(10) Course interest	**.31**	.13	.17	.12	.14	.10	.02	.05	**.27**	–	**.27**	**.24**	.15	**.24**
(11) Course motivation	**.30**	.10	.20	−.07	.00	−.14	.12	−.01	.07	**.29**	–	.18	.16	**.25**
(12) Pre-course knowledge	.22	.03	.04	**.29**	**.52**	−.04	.05	−.06	−.07	.03	.13	–	**.44**	**.54**
(13) Post-course knowledge	**.48**	.18	−.06	.20	.24	.01	**.31**	.04	.10	.03	**.30**	**.31**	–	**.77**
(14) Test average	**.51**	.19	.01	.18	.22	.03	**.33**	.01	.07	.17	**.47**	**.31**	**.87**	–

Note. Correlations for Section 1 (listwise *n* = 68) are presented below the diagonal; correlations for Section 2 (listwise *n* = 111) are presented above the diagonal. Coefficients in bold are statistically significant at *p* < .05.

**Table 3:** Correlations of Course Performance with ACT subtest scores.

	1	2	3	4	5	6
(1) Post-course knowledge	–	**.77**	**.23**	.17	**.29**	**.26**
(2) Test average	**.87**	–	**.50**	**.36**	**.40**	**.39**
(3) ACT English	**.32**	**.47**	–	**.59**	**.62**	**.53**
(4) ACT Mathematics	**.49**	**.49**	**.54**	–	**.42**	**.73**
(5) ACT Reading	**.36**	**.37**	**.72**	**.43**	–	**.63**
(6) ACT Natural Science	**.46**	**.46**	**.47**	**.65**	**.51**	–

Note. Correlations for Section 1 (listwise *n* = 70) are presented below the diagonal; correlations for Section 2 (listwise *n* = 111) are presented above the diagonal. Coefficients in bold are statistically significant at *p* < .05.

**Table 4:** Regression Analyses Predicting Test Average.

			Section 1				Section 2			
Model	Step	Predictor	Δ*R*^2^	β	*t*	*p*	Δ*R*^2^	β	*t*	*p*
1	1	ACT	.27	.52	5.01	<.001	.25	.50	6.10	<.001
2	1	*g*	.06	−.07	−0.52	.603	.07	−.11	−1.02	.310
	2	ACT	.21	.56	4.38	<.001	.19	.58	5.22	<.001
3a	1	*g*	.06	−.14	−1.07	.290	.07	−.12	−1.25	.215
	2	Pre-course know	.06	.23	2.12	.038	.24	.41	5.16	<.001
	3	ACT	.20	.54	4.35	<.001	.10	.43	4.20	<.001
3b	1	*g*	.06	−.06	−0.50	.617	.07	−.11	−1.01	.313
	2	Conscientiousness	.00	.05	0.43	.672	.00	.01	0.13	.900
	3	ACT	.21	.55	4.33	<.001	.18	.58	5.14	<.001
3c	1	*g*	.06	−.07	−0.56	.576	.07	−.11	−1.02	.311
	2	Dutifulness	.00	.07	0.62	.540	.01	−.04	−0.52	.601
	3	ACT	.21	.56	4.38	<.001	.18	.57	5.12	<.001
3d	1	*g*	.06	−.08	−0.60	.548	.07	−.10	−0.94	.350
	2	Cautiousness	.00	−.07	−0.69	.491	.01	.13	1.60	.113
	3	ACT	.21	.57	4.41	<.001	.19	.59	5.33	<.001
3e	1	*g*	.06	−.05	−0.42	.675	.07	−.13	−1.13	.261
	2	Self-efficacy	.01	.11	1.08	.284	.00	.05	0.62	.534
	3	ACT	.21	.56	4.41	<.001	.19	.59	5.22	<.001
3f	1	*g*	.06	−.07	−0.50	.621	.07	−.05	−0.44	.664
	2	Achiev. striving	.01	−.01	−0.06	.954	.05	−.16	−1.84	.068
	3	ACT	.20	.56	4.29	<.001	.16	.54	4.89	<.001
3g	1	*g*	.06	−.07	−0.55	.586	.07	−.10	−0.87	.385
	2	Self-discipline	.00	−.03	−0.29	.774	.01	−.06	−0.70	.487
	3	ACT	.21	.56	4.35	<.001	.18	.57	5.17	<.001
3h	1	*g*	.06	−.07	−0.52	.604	.07	−.11	−1.01	.313
	2	Orderliness	.00	−.01	−0.07	.949	.01	.02	−0.27	.792
	3	ACT	.21	.56	4.33	<.001	.18	.58	5.14	<.001
3i	1	*g*	.06	.00	0.01	.992	.07	−.11	−1.01	.315
	2	Openness	.11	.27	2.69	.009	.00	−.02	−0.28	.783
	3	ACT	.17	.51	4.12	<.001	.19	.58	5.21	<.001
3j	1	*g*	.06	−.07	−0.52	.608	.07	−.12	−1.04	.303
	2	Self-control	.00	.00	0.03	.976	.01	.02	0.21	.833
	3	ACT	.21	.56	4.34	<.001	.18	.58	5.13	<.001
3k	1	*g*	.06	−.07	−0.52	.604	.07	−.11	−0.98	.327
	2	Grit	.00	−.01	−0.07	.946	.00	−.03	−0.30	.767
	3	ACT	.21	.56	4.32	<.001	.19	.58	5.20	<.001
3l	1	*g*	.06	−.04	−0.35	.726	.07	−.08	−0.77	.442
	2	Course motivation	.21	.36	3.51	<.001	.07	.23	2.90	.004
	3	ACT	.11	.43	3.51	<.001	.17	.55	5.13	<.001
3m	1	*g*	.06	−.07	−0.52	.606	.07	−.10	−0.95	.344
	2	Course interest	.01	.00	0.01	.990	.05	.16	1.98	.051
	3	ACT	.20	.56	4.24	<.001	.16	.55	4.93	<.001

Note. β*s*, *t*s, and *p* values are presented for the full model; Δ*R*^2^ values are presented for each step of the model.

**Table 5:** Regression Analyses with ACT Subtests Predicting Test Average.

	Section 1				Section 2			
Predictor	*R* ^2^	β	*t*	*p*	*R* ^2^	β	*t*	*p*
English	.03	.28	1.73	.089	.07	.37	3.13	.002
Mathematics	.02	.21	1.45	.153	.00	.01	0.06	.953
Reading	.00	−.03	−0.22	.831	.00	.08	0.67	.504
Natural Science	.02	.21	1.48	.143	.01	.14	0.99	.323
**Overall Model**	.32			<.001	.28			<.001

Note. The *R*^2^ value for each subtest is the squared semi-partial *r*, reflecting unique variance explained.

**Table 6:** Parameter Estimates from SEM for the Mediation Models.

Mediator	Model Fit	*R* ^2^	*g* to PCK	ACT to PCK	*g* to M	ACT to M	M to PCK
No mediator	*χ*^2^(8) = 41.00 *p* < .001, RMSEA = .15, CFI = .86, NFI = .84	10.9%	β = .24 *p* = .008	β = .23 *p* = .023	--	--	--
Pre-course know.	*χ*^2^(11) = 45.79, *p* < .001, RMSEA = .13, CFI = .87, NFI = .84	18.1%	β = .11 *p* = .277	β = .22 *p* = .025	β = .44 *p* < .001	β = .01 *p* = .899	β = .31 *p* < .001
Conscientiousness	*χ*^2^(11) = 44.46, *p* < .001, RMSEA = .13, CFI = .86, NFI = .83	11.0%	β = .24 *p* = .007	β = .23 *p* = .023	β = .09 *p* = .319	β = .09 *p* = .374	β = −.02 *p* = .763
Dutifulness	*χ*^2^(11) = 45.27, *p* < .001, RMSEA = .13, CFI = .85, NFI = .82	11.0%	β = .24 *p* = .007	β = .23 *p* = .022	β = −.04 *p* = .691	β = −.10 *p* = .360	β = .03 *p* = .682
Cautiousness	χ^2^(11) = 43.93, *p* < .001, RMSEA = .13, CFI = .86, NFI = .83	11.2%	β = .25 *p* = .006	β = .22 *p* = .028	β = −.17 *p* = .062	β = .08 *p* = .467	β = .06 *p* = .401
Self-efficacy	*χ*^2^(11) = 44.25, *p* < .001, RMSEA = .13, CFI = .86, NFI = .83	11.9%	β = .24 *p* = .009	β = .25 *p* = .017	β = .01 *p* = .890	β = −.17 *p* = .113	β = .10 *p* = .174
Achiev. striving	*χ*^2^(11) = 46.03, *p* < .001, RMSEA = .13, CFI = .86, NFI = .83	11.3%	β = .26 *p* = .010	β = .21 *p* = .070	β = .28 *p* = .002	β = −.33 *p* = .006	β = −.07 *p* = .427
Self-discipline	*χ*^2^(11) =45.27 , *p* < .001, RMSEA = .13, CFI = .85, NFI = .82	11.1%	β = .25 *p* = .007	β = .22 *p* = .026	β = .14 *p* = .123	β = −.08 *p* = .435	β = −.04 *p* = .585
Orderliness	*χ*^2^(11) = 44.93, *p* < .001, RMSEA = .13, CFI = .86, NFI = .83	11.0%	β = .24 *p* = .007	β = .23 *p* = .023	β = .09 *p* = .305	β = .10 *p* = .332	β = −.02 *p* = .740
Openness	*χ*^2^(11) = 47.91, *p* < .001, RMSEA = .14, CFI = .84, NFI = .81	10.9%	β = .24 *p* = .008	β = .23 *p* = .026	β = .01 *p* = .922	β = .14 *p* = .191	β = .01 *p* = .890
Self-control	*χ*^2^(11) = 42.44, *p* < .001, RMSEA = .13, CFI = .87, NFI = .83	11.1%	β = .24 *p* = .008	β = .23 *p* = .022	β = .00 *p* = .97	β = −.16 *p* = .133	β = .04 *p* = .545
Grit	*χ*^2^(11) = 43.44, *p* < .001, RMSEA = .13, CFI = .86, NFI = .83	10.9%	β = .24 *p* = .008	β = .23 *p* = .023	β = .11 *p* = .225	β = .01 *p* = .895	β = .00 *p* = .966
Course motivation	*χ*^2^(11) = 43.40, *p* < .001, RMSEA = .13, CFI = .87, NFI = .84	14.0%	β = .24 *p* = .007	β = .19 *p* = .064	β = .02 *p* = .804	β = .21 *p* = .045	β = .18 *p* = .013
Course interest	*χ*^2^(11) = 43.47, *p* < .001, RMSEA = .13, CFI = .86, NFI = .83	10.9%	β = .24 *p* = .009	β = .23 *p* = .025	β = .17 *p* = .055	β = .14 *p* = .187	β = .01 *p* = .916

Note. PCK = post-course knowledge. M = mediator.

## Data Availability

De-identified data are publicly available on the Open Science Framework at https://osf.io/xen2h.

## Notes

1 Note that each “Step” represents a separate model. The focus of these analyses is on the incremental variance accounted for by the inclusion of an additional predictor in each Step.

2 Note that we use the term “mediation” here to refer to the indirect effect capturing the covariation between ACT scores, predictor/mediator variables of interest, and class performance. The purpose of these analyses is to determine whether the predictor variables of interest explain the relationship between the ACT and class performance. Upon examination of the direction of the arrows in Figure 1, it could be wrongly assumed that we are suggesting ACT scores have a causal influence on the mediators, which in turn predict class performance. This is not the case. We are not suggesting that, for example, ACT scores *cause* personality differences which in turn are causally related to class performance.

3 “Fairness”, refers not only to measurement bias (i.e., differential prediction across subgroups), but also to equity, accessibility, and the principles of universal design. As outlined in the *Standards for Educational and Psychological Testing* (Joint Committee 2018), fairness is a broad concept; we use the term in this context mainly to refer to issues of equity, such as differential access to scholarly opportunities, and the consequences of these inequities. For a review, see Woo et al. (2022).

## References

[B1-jintelligence-11-00009] Ackerman Phillip L., Kanfer Ruth, Dai David Y., Sternberg Robert J. (2004). Cognitive, affective, and conative aspects of adult intellect within a typical and maximal performance framework. Motivation, Emotion, and Cognition.

[B2-jintelligence-11-00009] (2017). ACT Technical Manual. http://www.act.org/content/dam/act/unsecured/documents/ACT_Technical_Manual.pdf.

[B3-jintelligence-11-00009] Butler Andrew C. (2010). Repeated testing produces superior transfer of learning relative to repeated studying. Journal of Experimental Psychology: Learning, Memory, and Cognition.

[B4-jintelligence-11-00009] College Scorecard Data. https://collegescorecard.ed.gov/data/.

[B5-jintelligence-11-00009] Costa Paul T., McCrae Robert R. (1992). Four ways five factors are basic. Journal of Personality and Individual Differences.

[B6-jintelligence-11-00009] Coyle Thomas R., Pillow David R. (2008). SAT and ACT predict college GPA after removing *g*. Intelligence.

[B7-jintelligence-11-00009] Coyle Thomas R., Snyder Anissa C., Richmond Miranda C., Little Michelle (2015). SAT non-g residuals predict course specific GPAs: Support for investment theory. Intelligence.

[B8-jintelligence-11-00009] Currie Janet, Thomas Duncan, Polachek Solomon W. (2001). Early test scores, school quality and SES: Longrun effects on wage and employment outcomes. Worker Wellbeing in a Changing Labor Market.

[B9-jintelligence-11-00009] Deary Ian J., Strand Steve, Smith Pauline, Fernandes Cres (2007). Intelligence and educational achievement. Intelligence.

[B10-jintelligence-11-00009] Donnellan M. Brent, Oswald Frederick L., Baird Brendan M., Lucas Richard E. (2006). The mini-IPIP scales: Tiny-yet-effective measures of the Big Five factors of personality. Psychological Assessment.

[B11-jintelligence-11-00009] Duckworth Angela Lee, Gross James J. (2014). Self-control and grit: Related but separable determinants of success. Current Directions in Psychological Science.

[B12-jintelligence-11-00009] Duckworth Angela Lee, Quinn Patrick D. (2009). Development and validation of the Short Grit Scale (GRIT–S). Journal of Personality Assessment.

[B13-jintelligence-11-00009] Ekstrom Ruth B., French John W., Harman Harry H., Derman Diran (1976). Kit of Factor-Referenced Cognitive Tests.

[B14-jintelligence-11-00009] Evans David E., Rothbart Mary K. (2007). Developing a model for adult temperament. Journal of Research in Personality.

[B15-jintelligence-11-00009] Finney Boylan Jennifer (2014). Save Us from the SAT. https://www.nytimes.com/2014/03/07/opinion/save-us-from-the-sat.html.

[B16-jintelligence-11-00009] Frey Meredith C., Detterman Douglas K. (2004). Scholastic assessment or g: The relationship between the scholastic assessment test and general cognitive ability. Intelligence.

[B17-jintelligence-11-00009] Goldberg Lewis R., Mervielde Ivan, Deary Ian, de Fruyt Filip, Ostendorf Fritz (1999). A broad-bandwidth, public domain, personality inventory measuring the lower-level facets of several five-factor models. Personality Psychology in Europe.

[B18-jintelligence-11-00009] Hambrick David Z., Chabris Christopher (2014). IQ Tests and the SAT Measure Something Real and Consequential. https://slate.com/technology/2014/04/what-do-sat-and-iq-tests-measure-general-intelligence-predicts-school-and-life-success.html.

[B19-jintelligence-11-00009] Hambrick David Z., Meinz Elizabeth J., Pink Jeffrey E., Pettibone Jonathan C., Oswald Frederick L. (2010). Learning outside the laboratory: Ability and non-ability influences on acquiring political knowledge. Learning and Individual Differences.

[B20-jintelligence-11-00009] Hartocollis Anemona (2019). SAT’s New ‘Adversity Score’ Will Take Students’ Hardships into Account. https://www.nytimes.com/2019/05/16/us/sat-score.html.

[B21-jintelligence-11-00009] Hayes Andrew F. (2009). Beyond Baron and Kenny: Statistical mediation analysis in the new millennium. Communication Monographs.

[B22-jintelligence-11-00009] Ingraham Christopher (2015). This Chart Shows How Much more Ivy League Grads Make than You. https://www.washingtonpost.com/news/wonk/wp/2015/09/14/this-chart-shows-why-parents-push-their-kids-so-hard-to-get-into-ivy-league-schools/?noredirect=on.

[B23-jintelligence-11-00009] Joint Committee (Joint Committee on the Standards for Educational and Psychological Testing of the American Educational Research Association, the American Psychological Association, and the National Council on Measurement in Education) (2018). Standards for Educational and Psychological Testing.

[B24-jintelligence-11-00009] Kane Michael J., Hambrick David Z., Tuholski Stephen W., Wilhelm Oliver, Payne Tabitha W., Engle Randall W. (2004). The generality of working memory capacity: A latent-variable approach to verbal and visuospatial memory span and reasoning. Journal of Experimental Psychology: General.

[B25-jintelligence-11-00009] Koenig Katherine A., Frey Meredith C., Detterman Douglas K. (2008). ACT and general cognitive ability. Intelligence.

[B26-jintelligence-11-00009] Kuncel Nathan R., Hezlett Sarah A. (2007). Standardized tests predict graduate students’ success. Science.

[B27-jintelligence-11-00009] Lee Woogul, Lee Myung-Jin, Bong Mimi (2014). Testing interest and self-efficacy as predictors of academic self-regulation and achievement. Contemporary Educational Psychology.

[B28-jintelligence-11-00009] Lorin Janet (2022). Why U.S. Colleges Are Rethinking Standardized Tests. The Washington Post.

[B29-jintelligence-11-00009] McCrae Robert R., Costa Paul T., Hogan Robert, Johnson John, Briggs Stephen (1997). Conceptions and correlates of openness to experience. Handbook of Personality Psychology.

[B30-jintelligence-11-00009] Richardson Michelle, Abraham Charles, Bond Rod (2012). Psychological correlates of university students’ academic performance: A systematic review and meta-analysis. Psychological Bulletin.

[B31-jintelligence-11-00009] Sackett Paul R., Kuncel Nathan R., Arneson Justin J., Cooper Sara R., Waters Shonna D. (2009). Does socioeconomic status explain the relationship between admissions tests and post-secondary academic performance?. Psychological Bulletin.

[B32-jintelligence-11-00009] Schmidt Frank L., Hunter John E., Outerbridge Alice N., Goff Stephen (1988). Joint relation of experience and ability with job performance: Test of three hypotheses. Journal of Applied Psychology.

[B33-jintelligence-11-00009] Schmitt Neal, Keeney Jessica, Oswald Frederick L., Pleskac Timothy J., Billington Abigail Q., Sinha Ruchi, Zorzie Mark (2009). Prediction of 4-year college student performance using cognitive and noncognitive predictors and the impact on demographic status of admitted students. Journal of Applied Psychology.

[B34-jintelligence-11-00009] Singh Kusum, Granville Monique, Dika Sandra (2002). Mathematics and science achievement: Effects of motivation, interest, and academic engagement. The Journal of Educational Research.

[B35-jintelligence-11-00009] Tangney June P., Boone Angie Luzio, Baumeister Roy F. (2004). High self-control predicts good adjustment, less pathology, better grades, and interpersonal success. Journal of Personality.

[B36-jintelligence-11-00009] Trapmann Sabrina, Hell Benedikt, Hirn Jan-Oliver W., Schuler Heinz (2007). Meta-analysis of the relationship between the Big Five and academic success at university. Journal of Psychology.

[B37-jintelligence-11-00009] Woo Sang Eun, LeBreton James M., Keith Melissa G., Tay Louis (2022). Bias, Fairness, and Validity in Graduate-School Admissions: A Psychometric Perspective. Perspectives on Psychological Science.

[B38-jintelligence-11-00009] Yenilmez Ayse, Sungur Semra, Tekkaya Ceren (2006). Students’ achievement in relation to reasoning ability, prior knowledge and gender. Research in Science & Technological Education.

[B39-jintelligence-11-00009] Zachary R. A., Shipley W. C. (1986). *S*Note that each “Step” represents a separate model. The focus of these analyses is on the incremental variance accounted for by the inclusion of an additional predictor in each Step. Shipley Institute of Living Scale: Revised Manual.

